# Failure of dual radius hydroxyapatite-coated acetabular cups

**DOI:** 10.1186/1749-799X-3-35

**Published:** 2008-08-07

**Authors:** Fabio D'Angelo, Mauro Molina, Giacomo Riva, Giovanni Zatti, Paolo Cherubino

**Affiliations:** 1 Department of Orthopaedics and Traumatology, University of Insubria, Varese, Italy

## Abstract

**Introduction:**

Many kind of hydroxyapatite-coated cups were used, with favorable results in short term studies; it was supposed that its use could improve osteointegration of the cup, enhancing thus stability and survivorship. The purpose of this study is to analyze the long term behavior of the hemispheric HA coated, Dual Radius Osteonics cup and to discuss the way of failure through the exam of the revised components and of both periacetabular and osteolysis tissue.

**Materials and Methods:**

Between 1994 and 1997, at the Department of Orthopedic Sciences of the Insubria University, using the posterolateral approach, were implanted 276 Dual Radius Osteonics^® ^in 256 patients, with mean age of 63 years.

**Results:**

At a mean follow-up of 10 years (range 8–12 years), 183 cups in 165 patients, were available for clinical and radiographical evaluation. 22 Cups among the 183 were revised (11%). The cause of revision was aseptic loosening in 17 cases, septic loosening in one case, periprosthetic fracture in another case, osteolysis and polyethylene wear in two cases and, finally, recurrent dislocations in the last one. In the remaining patients, mean HHS increased from a preoperative value of 50,15 to a postoperative value of 92,69. The mean polyethylene wear was 1,25 mm (min. 0,08, max. 3,9 mm), with a mean annual wear of 0,17 mm. The mean acetabular migration on the two axis was 1,6 mm and 1,8 mm. Peri-acetabular osteolysis were recorded in 89% of the implants (163 cases). The cumulative survivorship (revision as endpoint) at the time was 88,9%.

**Conclusion:**

Our study confirms the bad behavior of this type of cup probably related to the design, to the method of HA fixation. The observations carried out on the revised cup confirm these hypotheses but did not clarify if the third body wear could be a further problem. Another interesting aspect is the high incidence of osteolysis, which are often asymptomatic becoming a problem for the surgeon as the patient refuses the possibility of a revision.

## Introduction

Cementless press-fit fixation of the acetabular component in total hip arthroplasty (THA) has been used for more than two decades. A variety of shell designs, locking mechanisms, fixation surfaces, supplemental fixation, and bearing surfaces have been used. Cementless fixation on the acetabular side requires an initial tight interlock between the implant and the reamed acetabulum followed by secondary fixation through osteointegration at the bone-implant interface achieved by means of bone ingrowth or ongrowth into the substrate.

Hydroxyapatite, an osteoconductive material shown to improve bone ingrowth or ongrowth, has been applied to femoral and acetabular components of differing designs with varying results. On the femoral side the aseptic revision rate has been excellent with a mechanical failure rate of less than 1% at 10-to 13-year follow-up [1-3]. On the acetabular side, the topic is debated with different failure rate in relation to the different acetabular component designs [3].

The aim of this study is to analyze the long term behaviour of a HA covered hemispheric implant and to discuss the mode of failure by the examination of those cup revised and of the tissues around the implant and in the osteolysis.

## Materials and Methods

Between 1994 and 1997, at the Department of Orthopedic Sciences of the Insubria University, using the posterolateral approach, a series of 276 hydroxyapatite-coated hemispheric cups were implanted, in 256 patients. There were 160 women (63%) and 96 man (37%). Mean age at the time of surgery was 63 years (range, 23 to 86 years). 20 patients, 10 women and 10 men, underwent a bilateral arthroplasty.

The primary diagnosis was osteoarthritis in 170 hips (61,5%), developmental dysplasia in 66 (23,9%), avascular necrosis in 5 (1,9%), secondary inflammatory arthritis in 25 (9,1%), secondary osteoarthritis due to acetabulum fracture in 2 (0,8%) and femur neck fractures in 8 (2,8%) hips (Tab. 1)

**Table 1:** Diagnosis at the time of primary surgery

***Diagnosis***		***Implants***	***%***
		
*Primary Coxarthritis*		170	61,5%
*Secondary Coxarthritis*	Dysplasia	66	23,9%
	
	Avascular necrosis	5	1,9%
	
	Inflammatory	25	9,1%
	
	Acetabular Fractures	2	0,8%

*Femur Neck Fractures*		8	2,8%

***Tot.***		**276**	**100%**

All patients received the same cementless acetabular cup (Dual Radius Osteonics^®^, Osteonics^®^, Allendale, NJ). This acetabular component was a HA-coated smooth hemispheric cup.

**Figure 1:**
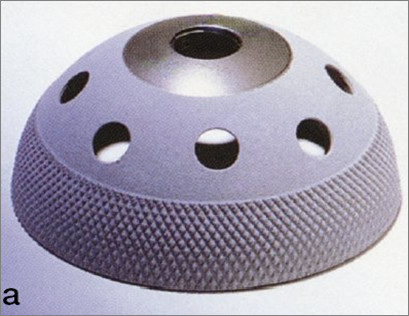
***Omnifit *HA-Coated cup.**

The press-fit Omnifit PS (peripheral self-locking) Dual Radius Osteonics^® ^(Figure 1) cup was a hemispheric design cup, with a metallic shell metallic shell of titanium alloy (Ti-6A1-4V) with multiple holes for additional screw fixation; the implant has a knurled surface machined around the periphery to a depth of 200 μm to improve the security of the press-fit achieved at the time of the surgery. The unused holes were not plugged.

According to the manufacturer, the surface was plasma sprayed to give a 50 μm covering of hydroxyapatite of > 97% purity, < 3% porosity, > 70% crystallinity and with a Ca/P ratio of 1.7.

Tensile bond strength is greater than 65 MPa and the fatigue bond strength is greater than 107 tensile/tensile cycles under 8.3 MPa.

The polyethylene inserts were beveled at 10° to the plane of the opening of the shell. A metallic wire connected to 4 hooks in the shell secured the liner. The PE inserts, made from base resin GUR 415, had been γ-irradiated and stored in air.

The cup was implanted according to "press-fit" surgical technique, after reaming the acetabular bone with a hemispheric cutter, 1 or 2 mm smaller than the measure of the implant. In case of poor bone quality, additional fixation of the cups to bone was achieved by placing 1 or 2 screws into the ilium through the dome holes provided in the shell.

In 210 cases, these cups were associated with an uncemented stem (25 Conus Protek^®^, 72 Omnifit Osteonics^®^, 16 Versys Zimmer^®^, 97 ZM Allopro^®^), in the remaining 65 cases with cemented stem (21 Chesi Protek^® ^e 44 Harris Galante Zimmer^®^). The heads assembled were metallic in 263 patients and ceramic in the other implants; the diameters of these heads were 28 mm in most cases, with the exception of three implants, in which one 32 mm head and two 22 mm were used.

In all patients a second generation cephalosporin was used as prophylaxis for infections. All patients received a four-week course of low molecular heparin as prophylaxis for venous thromboembolism and a three week course of indomethacin as prophylaxis for heterotopic ossification. All patients walked with full weight-bearing with two crutches for the first month and then the crutches was removed one by one in the consecutive two months.

Patients were assessed clinically using the Harris Hip Score (HHS) to determine the level of function pre-operatively and at the final follow-up. Post operative scores of 90 points or more were graded as excellent, 80–89 points as good, 70–79 points as fair and less than 69 points as poor [4].

At the time of follow-up, AP views of the hip and pelvis were taken with a true lateral view of the hip and compared with those taken at the six first months postoperatively. They were converted to digital files for storage and later analysis using a scanner (Epson Scan 1640 XL^®^, Seiko Epson Corporation, Japan).

**Figure 2:**
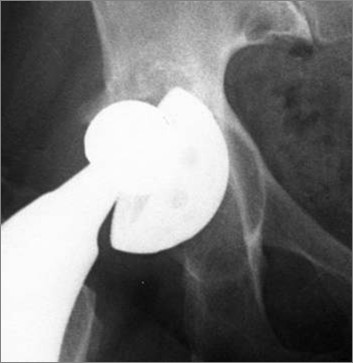
AP view of a completely loosed cup.

Any visible migration of the acetabular component radiolucent lines, osteolysis and polyethylene wear were measured with the commercially available software "Polyware"^® ^and with digital caliper "Sigma scan"^® ^[5,6] (Fig. 2).

Any migration was evaluated by measuring the vertical and the horizontal distance from the acetabular cup centre to the radiological "U" (Fig. 2). The acetabular inclination was reckoned measuring the angle between the tangent to the U and the tangent to the cup open side. A variation than 5 degrees was considered significant [7].

Radiolucent line means a line of increased Rx transparency next to acetabulum, delimited by a sclerotic line. Any radiolucency 2 mm or greater was considered significant [8,9]

Osteolysis means an area of well delimited reduced bone density independently from dimensions. The position of both was stated according to Delee and Charnley areas [8].

Acetabular interface stability was determined using the criteria described by Capello and Kawamura [10-12]:

• *Stable by bone ingrowth: *components with either no radiolucent lines or radiolucent lines in one or two zones only, and with no measurable migration.

• *Stable by fibrous ingrowth: *components with radiolucent lines in all three zones, and with no measurable migration.

• *Unstable: *cups that migrated 3 mm or more and showed radiolucent lines in all 3 zones.

Paired T-Test was used to compare the HHS calculated before and after the operation with the statistical significance set at p < 0.05.

**Figure 3:**
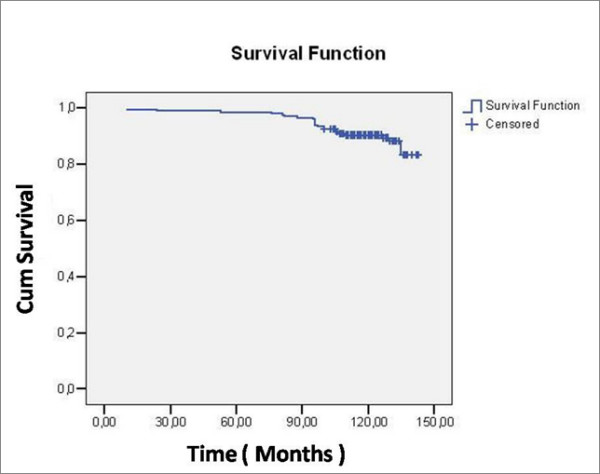
Kaplan-Meyer survival curve for end point for cup revision.

Kaplan-Meier survivorship analysis was performed on the cohort of 199 hips (Table 2 – Fig. 3), because 77 implants were completed lost ad follow-up using cup revision as end-point (16 patients in serious clinical condition, unable to come to clinical evaluation, were included in Kaplan-Meier survivorship analysis because they didn't undergo revision surgery).

**Table 2:** Case processing summary. The end-point is the cup revision.

Total N	N of Events	Censored	
		
		N	Percent
199	22	177	88,9%

In case of revision of the cup, a further evaluation was performed.

The metal-back and the polyethylene were examined under SEM-FEG XL 30 (Philips) scanning microscope, according to SE procedure, upon previous gold metallization (Av) with Sputter K250 (EMiteh). A microanalysis with EDAX microanalyzer mounted on SEM-FEG XL-30 was performed on the same materials.

The periacetabular tissue and the bone of the area of osteolysis were smashed to about 2 × 3 mm fragments, fixed in a Karnowski solution, washed in 0.1 M saccharose cacodylate buffer, dehydrated in an alcohol rising scale, and finally included in paraffin envelope. The sections obtained with Leica microtome were assembled on a slide and stained with haematoxylin – eosin.

The exam was performed with Nikon Eclipse 600 microscope, using polarized light in order to find polyethylene debries.

## Results

### Clinical results

At an average follow-up of 10 years (range, 8 to 12), we completely lost seventy-five patients, two of them with bilateral arthroplasty. 51 patients were not reliable and 24 were died for causes not related with the operation.

Moreover 16 patients were in serious clinical conditions for associated pathologies and so unable to come the control. These 16 patients were assessed by telephone with Harris Hip score; they all referred to be satisfied of their joint and were included in HHS and Kaplan-Meyer survivorship analysis, which was therefore performed of cohort of 199 patients.

Finally, these sixteen were excluded from other evaluations, for a final number of 183 hips in 165 patients.

The average Harris hip score increased from 50,15 points (range, 17 to 92 points) preoperatively to 92,69 points (range, 50 to 100 points) at the time of final follow-up. The difference between the pre-operative and final HHS was statistically significant according to the t test (p < 0.05). The clinical outcome of 131 hips (71,6%) was graded excellent, 26 (14,2%) good, 18 (9,8%) fair and 8 (4,4%) poor.

In the post-operative period, among the full cohort 20 complications (7,2%) were recorded. Seven of them were general ones (1 pulmonary embolism, 1 acute renal insufficiency, 1 myocardial ischemia, 1 bleeding duodenal ulcer, 2 deep venous thrombosis, 1 urinary tract infection), 1 (0,4%) femoral nerve neurotmesis, 6 (2%) problems related to the surgical wound (5 suprafascial haematomas, 1 dehiscence and 1 superficial infection), which required another surgical procedure.

There were five (2%) early dislocations, all of which were treated with closed reduction and restriction of weight bearing for four weeks.

22 Cups among the 183 were revised (12%). The revision cause was aseptic loosening in 17 cases, septic loosening in one case, periprosthetic fracture in another case, osteolysis and polyethylene wear in two cases and, finally, recurrent dislocations in the last hip. Survivorship analysis showed that survival of the cup was 88.9% at 12 years with 95%confidence interval (Fig. 3).

### Radiological results

Examination for radiolucent lines showed lines larger than 2 mm in 15 implants (8,1%), but only in one case, they influenced all three Charnley areas. This cup was considered to be probably loosed, as it did not reveal any migration. In two cases, they influenced areas 2 and 3, in 1 only area 1 and in the remaining eleven only area 3.

The mean polyethylene wear was 1,25 mm (min. 0,08, max. 3,9 mm), with a mean annual wear of 0,17 mm.

The mean acetabular migration on the two axes was 1,6 mm and 1,8 mm. Only in 11 implants (6%) an  acetabular migration greater than 3 mm was recorded. At six month follow-up, the mean acetabular inclination angle was 48° (min 36°, max 70°). At the final control a 3,9° medium variation (min 0°, max 6.5°) was recorded. Only in two patients (4 implants) an angle variation greater than 5° was recorded.

**Figure 4:**
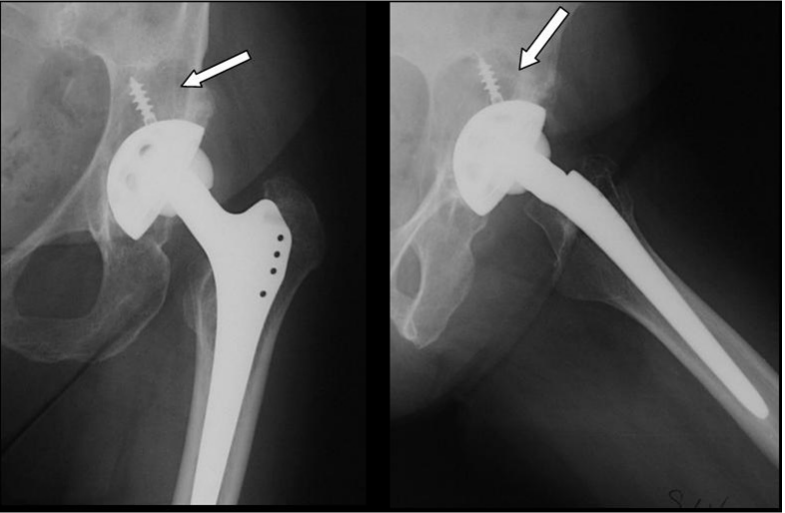
AP and lateral view show the large osteolysis (arrow) and the PE wear.

Periacetabular osteolysis was recorded in 89% of the implants (163 cases). Most of them, were located in Charnley areas number 2 and 3, in 8 implants (4,3%) they were located in all areas. The mean osteolysis area was 773 mm^2 ^in area 1, 489 mm^2 ^in area 2 e 151 mm^3 ^in area 3 (Fig. 4).

In 128 implants (70%) osteolysis were also recorded in the proximal femur (greater trochanter and calcar).

### SEM observation and histological results in case of revised cups

**Figure 5:**
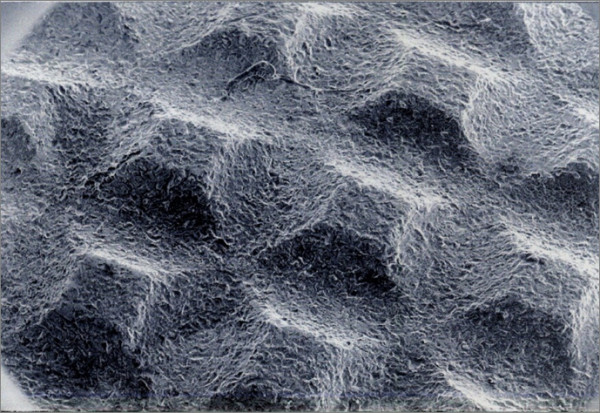
SEM Image of the surface of a removed cup.

**Figure 6:**
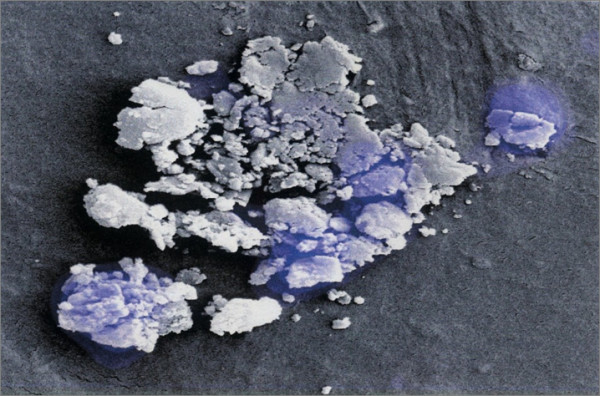
SEM Image of the remains on the polyethylene insert.

The SEM analysis of the acetabular cups allowed us to point out the completed HA disappearance from the metal back. Moreover, both on the internal and the external surface of the polyethylene liner, we observed many remains (Fig. 5 and Fig. 6).

**Figure 7:**
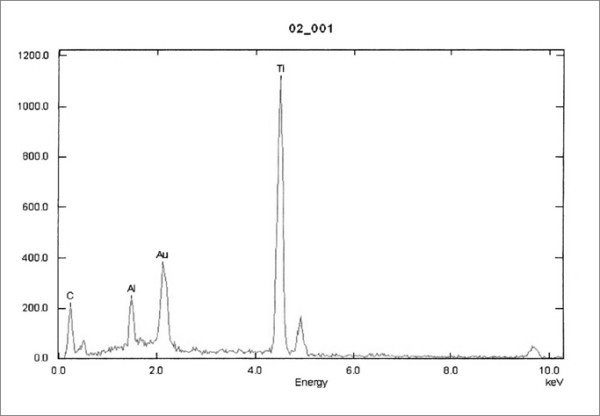
Cup's Microanalysis shows the absence of Ca and P.

**Figure 8:**
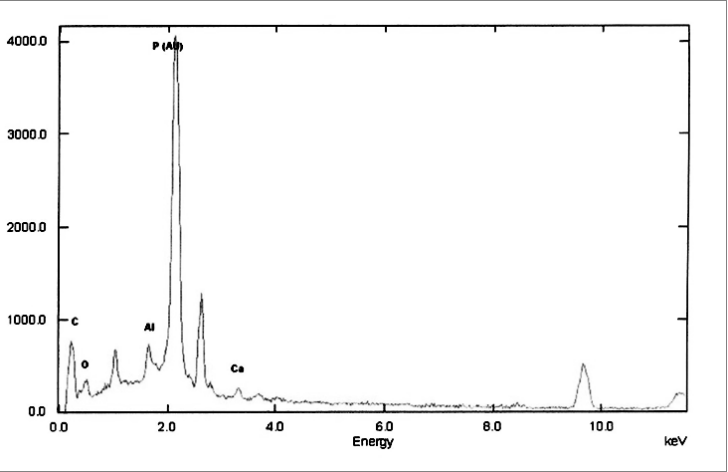
Remains microanalysis shows the presence of high amount of Ca and P.

Cup’s microanalysis showed low quantities of CA and P, main components of HA covering, besides other metallic elements such as Al Ti, C, O were found (Fig. 7). In the remains, we found a much higher concentration of Ca and P and low concentration of metallic elements (Fig. 8).

**Figure 9:**
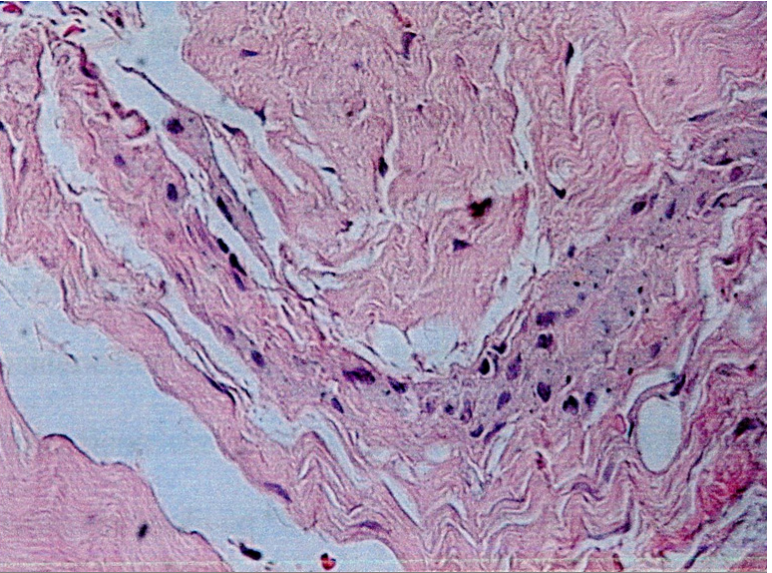
The light microscopy of the tissue inside an osteolytic shows the presence of hystiocytes, with cytoplasmatic inclusions (E-E stain).

The light microscopy of the osteolysis pointed out the presence of fibrous tissue with cell with many cytoplasmatic inclusions (Fig. 9).

## Discussion

With the spreading use of total hip arthroplasty, the number of revision for aseptic loosening is growing year by year; unfortunately the clinical results of the revisions are definitely worse than the first implants [13].

These remarks led research to develop several systems of fixation, which could warrantee a longer survivorship of the implant, leaving a sufficient bone stock for revision. Particular interest was devoted to hydroxyapatite (HA), which could be fixed to the metal surfaces of the components using different techniques [14], specially plasma spray one.

HA coatings have been shown to induce strong union with bone and to promote early stable fixation of the implant in an animal study [15], in a human retrieval study [16] and in early-term clinical follow-up studies [17-19]. So, it was hypothesized that the use of HA coverings could enhance biologic fixation of the implants, improving thus the longevity after midterm follow-up.

Although good medium and long term results with HA coated femoral stems have been reported [20,21], the use of HA coating on smooth hemispheric acetabular components does not seem as successful as in femoral ones [9,10,18,20-24].

Some authors reported satisfactory short term results using HA coated smooth hemispheric implants, noticing a reduction of cup migration and of periacetabular radiolucent lines [25-27]. In a multicentric study, D'Antonio et al. reported that, at two years follow-up, in a cohort of 320 HA coated cups, only three patients showed a significant migration, but none required a revision [26]. However, these initial encouraging results were not confirmed in mid and long term follow-up: poor results have been reported with HA-coated smooth press-fit cups from different manufacturers, with a revision rate ranged from 20% to 30%, after 7 to ten years follow-up [9,23,24,28-30]. Recently, Kim et al. reported poor results with the same cup of our study after midterm follow-up with a 13% of revision rate and 60.5% survival at 8 years with any revision as end points. In our study the rate of revision at an average follow-up of 10 years was 12%, but we noticed a higher rate of osteolysis, which interested both the cup and the proximal femur (respectively 89% of the cups and 70% of the stems). In literature the rate of osteolysis range from 28% to 66% [23,24]. This date could be partially explained with our longer follow-up. The periacetabular radiolucent lines incidence is comparable to the one found in other studies on HA coated cups; even the location of the line is mostly in the Zone 3 [9,23,27].

Only in 4 implants we reported variation of the acetabular angle higher than 5°, compatible with implant loosening according to the limits founds in literature [7]. In these patients, the angle variation was associated by linear migration of 0,3 mm, 1,3 mm, 1,8 mm and 1,4 mm. Anyway, in none the radiographic pattern was related to a low clinical evaluation (HHS 100, 97, 97, 90). It has been observed that all these four patients were in origin affected by dysplasia.

The polyethylene wear was slightly higher in our study than the one found in literature for HA coated cup with the same follow-up [9,31,32].

There are several possible reasons for failure of the HA-coated smooth hemispheric acetabular cups used in literature [33,34]. Manley et al. [23] evaluated 377 patients (428 hips) with a porous coated, press-fit acetabular cup, an HA-coated threaded screw-in cup, or one of two similar designs of HA-coated press-fit cups after an average of 7,9 years of follow-up. In this study, the probability of revision due to aseptic loosening was significantly greater for the HA-coated press fit cups, than for the HA-coated threaded cups or the porous-coated, press-fit cups (p < .001 for both comparisons). The HA-coated threaded cups and the porous coated press-fit cups continued to perform well more than 5 years after the operation.

The unsatisfactory results on the acetabular component suggest that in the specific biomechanical environment of the acetabulum, physical interlocking between the cup and the supporting bone beneath it may be a prerequisite for long-term stability; thus cup design is very critical for its performance [35,36]. Therefore, despite the good short term results with HA-coated press-fit cups (2–3 years), fatigue failure between the metal surface and the HA coating, arising in response to prolonged distractional stress medially imposed by the patient's activity, was thought to be responsible for the separation of the socket from the bone in the case of press-fit cups in the long term [24,37]. In other words, continued application of physiologic loads, especially tension and torsion, will cause motion and distraction between the acetabular components and the osseous structures beneath it, and progressive loosening at the interface and failure of fixation may occur. Initial stability dependent on a press fit and screws will necessarily fail [38]. The HA-coated threaded cups achieved sufficient bony and/or soft tissue interlock to resist the force load on the acetabular cup, whereas the HA-coated smooth hemispheric acetabular cups in many cases did not [9,39,40].

In HA-coated implants, one of the most important events occurring at the bone-implant interface is the resorption of the HA coating, also called "degradation or coating loss", sometimes with the presence of HA particles. Although it is essential for the establishment of bone-implant bonding, this has been one of the main concerns for the durability of the HA-coated implants.

Some studies have shown resorption of HA coatings up to 2 years after implantation [41-43] and a complete loss of a 60-mm-thick HA coating after 4 years [44].

Therefore, the long-term durability of the fixation enhanced by the HA coating is questionable [45,46]. Direct contact of bone trabeculae with the surface of the implant after degradation of the HA coating is dependent on implant material, texture, and design. Application of an HA coating to an implant with a smooth surface increases the risk of delamination of the coating compared with its application to a porous surface [46,47]. Resorption of the HA may cause micromotion with an increase in shear stresses, resulting in delamination of the HA, especially on the medial side of the cup.

An unacceptable accelerated polyethylene wear rate and high prevalence rate of pelvic osteolysis is described. Some authors suggested that HA particles could move and cause third-body abrasive wear, which subsequently could cause accelerated polyethylene wear and development of osteolysis [48,49].

The use in our department of a protocol for the examination of the retrieved implant and the bone-implant interface, give us the possibility pointed out something about the mechanism of failure.

The SEM examination of the cups showed the complete disappearance of the coating, as observed in other studies [44], and the complete absence of bone ongrowth. No HA particles were found on polyethylene and the microanalysis of the waste on the liner pointed out not only Ca e P, but also other elements such as Ti, Al, C, O, which can be decay products also of the metallic alloys forming the metal back and the screws. Therefore, it is impossible to assert with certainty HA may cause increased polyethylene wear.

**Figure 10:**
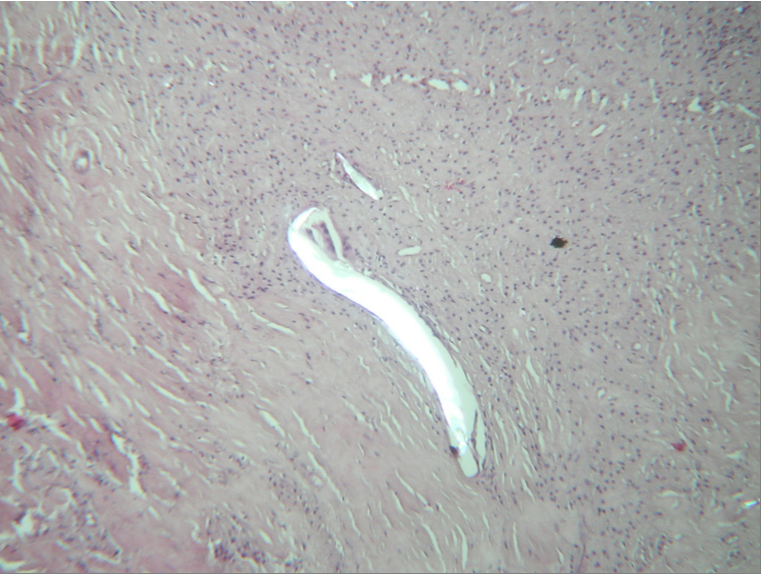
The light microscopy of the neocapsule shows the typical foreign body reaction to debris. The polarised light confirms that the debris are polyethylene as they are birefringent.

Many polyethylene debris were found in periacetabular tissue, using polarized light microscopy (Fig. 10).

Some authors believe that the incremented rate of osteolysis could be attributed to the fretting between the screws and the dome holes [50,51]: we can't confirm this hypothesis, because no association between the use of screws and both the presence and the dimension of osteolysis were found (p <*0,05*). Manley himself had stated in his study [23] that the dome hole could not considered a way of passage of wear of polyethylene.

The most interesting aspect of our study is the discordance the clinical and X-Ray results.

In spite of the incidence of osteolysis, most patients are absolutely asymptomatic and satisfied with their life quality. These bone rarefaction areas do not weaken the mechanical stability, but being progressive [9], when the revision is performed, we may risk to face such poor bone-stock as to spoil the result of revision operation. Thus, revision rate is lower than other study, as it's very difficult to give such indication in asymptomatic patients.

## Conclusion

At the end we can assert that in spite of the spreading of non cemented cups, we have not yet found the final solution for a long time of the implant, capable to guarantee a good bone stock for eventual quite safely revision.

The HA coatings applied on smooth hemispheric cups, even if they were shown to be able to speed up and make the bone prosthesis link more solid in the short period, imply a high risk of complication (osteolysis, wear, loosening, etc.) in the long period, probably connected with the inevitable material decay process.

It has not yet been proved with certainty that osteolysis increase is due to the third body wear; in fact we could make reference to many other factors, such as the cup design, the number of holes at the dome, the number of the screws, on which there are many discordant opinions in literature.

Finally, we have to consider the not little problem of the right timing of revision to prevent excessive bone loss, in patients probably hard to convince, because asymptomatic.

## Competing interests

The authors declare that they have no competing interests.

## Authors' contributions

FD conceived the study, and participated in its design, coordination and drafted the manuscript. MM and GR both carried out the clinical and radiological examination of all cases. GR also performed the computer acquisition of all the data and the statistical analysis. GR and PC both performed the surgery, as senior surgeon. All authors read and approved the final manuscript.
